# Mutation accumulation under UV radiation in *Escherichia coli*

**DOI:** 10.1038/s41598-017-15008-1

**Published:** 2017-11-06

**Authors:** Atsushi Shibai, Yusuke Takahashi, Yuka Ishizawa, Daisuke Motooka, Shota Nakamura, Bei-Wen Ying, Saburo Tsuru

**Affiliations:** 1 Graduate School of Information Science and Technology, Osaka University, 1-5 Yamadaoka, Suita, Osaka, 565-0871 Japan; 2 Department of Infection Metagenomics, Genome Information Research Center, Research Institute for Microbial Diseases, Osaka University, 3-1 Yamadaoka, Suita, Osaka, 565-0871 Japan; 3 Graduate School of Life and Environmental Sciences, University of Tsukuba, 1-1-1 Tennoudai, Tsukuba, Ibaraki, 305-8572 Japan; 4 Universal Biology Institute, School of Science, The University of Tokyo, 7-3-1 Hongo, Bunkyo-ku, Tokyo, 113-0033 Japan

## Abstract

Mutations are induced by not only intrinsic factors such as inherent molecular errors but also by extrinsic mutagenic factors such as UV radiation. Therefore, identifying the mutational properties for both factors is necessary to achieve a comprehensive understanding of evolutionary processes both in nature and in artificial situations. Although there have been extensive studies on intrinsic factors, the mutational profiles of extrinsic factors are poorly understood on a genomic scale. Here, we explored the mutation profiles of UV radiation, a ubiquitous mutagen, in *Escherichia coli* on the genomic scale. We performed an evolution experiment under periodic UV radiation for 28 days. The accumulation speed of the mutations was found to increase so that it exceeded that of a typical mutator strain with deficient mismatch repair processes. The huge contribution of the extrinsic factors to all mutations consequently increased the risk of the destruction of inherent error correction systems. The spectrum of the UV-induced mutations was broader than that of the spontaneous mutations in the mutator. The broad spectrum and high upper limit of the frequency of occurrence suggested ubiquitous roles for UV radiation in accelerating the evolutionary process.

## Introduction

Mutations, changes in the genetic code, are an essential source of variation for evolutionary processes^1,2^. Therefore, understanding the mutational properties of mutations is a fundamental task in evolutional biology^3^. The mutation spectrum is a property that affects the probability of each mutation (such as a transition from AT to GC), since it affects the fitness distribution of mutations. For instance, the emergence probability of mutants resistant to an antibiotic depends on the mutational spectrum^4^. That is, if a given mutation (e.g. AT to GC) is frequent and can favour resistance to a certain drug, the mutation cannot always effectively confer resistance to other drugs. Mutation rate is also a key property since it dictates the tempo of evolution. Higher mutation rates accelerate the speed of adaptive evolution^5^ as well as that of neutral evolution^4,6^. These lines of evidence strongly support the significance of understanding these mutational profiles.

Consistent with this importance, many studies have explored the rate and spectrum of mutations for different organisms^7^. These studies revealed unique mutational profiles at the genomic level and their contributions to evolutionary processes. For example, recent studies identified that the rate and spectrum of mutations in *Escherichia coli* varied among wild types and mutator strains^3,4,6,8^. Mutator strains exhibit higher spontaneous mutation rates due to intrinsic causes such as defective mutations in inherent error correction machineries (mismatch repair and proofreading) and arise frequently under both natural and laboratory conditions. The rate and spectrum of mutations define the roles of mutators in the adaptive evolution of a population^4,5^.

Contrary to these extensive studies on the spontaneous mutability of cells on the genomic scale, the profiles of mutations coupled with extrinsic factors are relatively unclear. It is well accepted that environmental mutagenic factors such as UV radiation and harmful chemicals increase mutation rates, and they must be considered to understand evolutionary processes in natural and clinical habitats. Therefore, it is important to assess both intrinsic and extrinsic contributions at the genomic level to gain a comprehensive understanding of the mutational process^3^. In addition, mutagens are also considered a tool that can be used to adjust mutation rates artificially for many applications^9,10^. The accurate identification of the mutational profiles of mutagens is necessary to control mutation speed more precisely and predictably. Most studies that have assessed the mutational profiles of mutagens were based on fluctuation tests with a few reporter loci on the genome^11^. This approach is conventional but inaccurate for assessing the representative profiles of the whole genome^3^. Moreover, the mutational profiles, mutation rate, and mutational spectrum can be very different between the restricted reporter loci and whole genome^6,8,12^. These lines of evidence indicate the importance of capturing the mutation profiles of mutagens over the genome.

Here, we explored the mutation profiles of UV radiation on the genomic scale. UV radiation is a standard mutagen, and its mutability has been studied extensively due to its ubiquity in nature and convenience of handling. In addition, UV is also applied in many fields such as UV disinfection technology in clinical microbiology^13^ and evolution engineering in the biotechnology^9,14^. Irradiation with UV radiation induces thymine dimer lesions in DNA sequences^15^. The unrepaired lesions increase the rate of replication errors, i.e. mutations. We performed evolution experiments using *E. coli* under periodic UV radiation for 28 days. Whole genome sequencing revealed the number and spectrum of the accumulated mutations. The spectrum was broad and similar to that of the spontaneous mutations of the wild type. The rate of the UV-induced mutations could become greater than the spontaneous mutation rate of the popular mutator strains with deficient mismatch repair processes. By keeping the number of cells after UV exposure constant at a lower level, the UV-induced mutation rates could be kept consistent regardless of the differences in the exposed UV dose, different viabilities against UV, and/or different spontaneous mutation rates. The broad spectrum and high upper limit of the speed in mutability of UV suggested ubiquitous roles of UV radiation in accelerating the evolutionary process.

## Results

### Viability and mutation probability in response to UV exposure in *E. coli*

UV radiation is a toxic mutagen and was expected to decrease the viability of the cells but also increase the probability of the emergence of mutants. We used three strains of *E. coli* to confirm how these standard actions of UV radiation affected them regarding inherent mutability, spontaneous mutation rate in the absence of UV exposure, and UV sensitivity. One was *E. coli* MDS42 with a proficient error correction system that was used as a standard control as was denoted Co. Two mutator strains, *ΔS* and *ΔHSB*, were constructed from Co by deleting the genes involved in error correction (*ΔmutS* for *ΔS* and *ΔmutH*, *ΔmutS*, and *ΔuvrB* for *ΔHSB*). The *mutS* and *mutH* genes are involved in the mismatch repair system, so the deletion of either/both genes increase the mutation rate as demonstrated previously^8^. The *uvrB* gene is one of the genes involved in the nucleotide excision repair function. This function has a role in repairing genomic DNA lesions mainly caused by UV radiation. Defects in *uvrB* reduce native UV resistance^16,17^. Therefore, *ΔHSB* was designed to be a UV-sensitive mutator strain.

**Figure 1:**
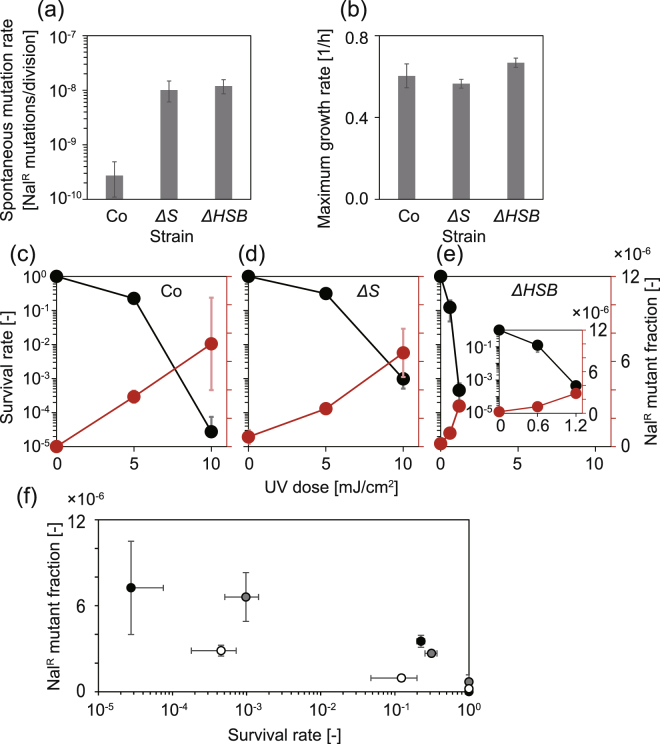
Mutability and viability of ancestral strains in response to UV exposure. (**a**) The spontaneous mutation rates of three strains before the evolution experiment. The error bars represent 95% confidential intervals calculated with the MSS method^26^. (**b**) The maximum growth rates of the ancestral strains. The error bars represent standard deviations (n = 60). (**c–e**) Effects of UV dose on the survival rates (black circles) and the Nal^R^ mutant fractions among the survivors (red circles) for the ancestral strains (Co (**c**), *ΔS* (**d**), and *ΔHSB* (**e**)). The error bars represent standard deviations (n = 3). (**f**) Scatter plots of survival rates and Nal^R^ mutant fractions of the ancestral strains (Co (black circles), *ΔS* (grey circles), and *ΔHSB* (white circles)). The values were replotted from (**c–e**).

First, we confirmed the increase in the spontaneous mutation rate of the mutators from Co. A fluctuation test based on a mutation resulting in resistance to nalidixic acid (Nal^R^) verified that the two mutators had about 40-times higher mutation rates compared to that of Co (Fig. 1a). These mutator strains exhibited only a slight difference in the growth rate (Fig. 1b, ANOVA, *F*(2,177) = 109, *p* < 0.05). These comparable rates were consistent with a previous study which reported that the growth rate decreased notably as the mutation rate increased beyond 100-fold of that of the wildtype^8^. Next, we confirmed the effects of UV exposure as a mutagen on the viability and mutability of the three strains (Fig. 1c–e). As expected, the survival rate, the viable fraction of the cell population, decreased as the UV dose increased for all strains. In contrast, the Nal^R^ mutant fraction increased. That is, the viability and mutability were negatively related (Fig. 1f). We found that *ΔHSB* was more susceptible to UV exposure in terms of reduced viability compared to the other strains, consistent with the lack of *uvrB* (Fig. 1e, black circles). In contrast, the Nal^R^ mutant fraction of this strain increased markedly more in response to the lower UV dose (red circles). When considering the equivalent mutation rates between the two mutator strains in the absence of UV exposure, the UV-sensitive viability of *ΔHSB* is due to its high, and specifically lethal, mutant production rate per UV dose. Furthermore, these results suggested that the UV-induced mutation rate could be kept at a high level by simply keeping the survival fraction constant regardless of different doses of UV exposure, different sensitivities to UV exposure, and/or different spontaneous mutation rates.

### Evolution experiment with UV exposure

**Figure 2:**
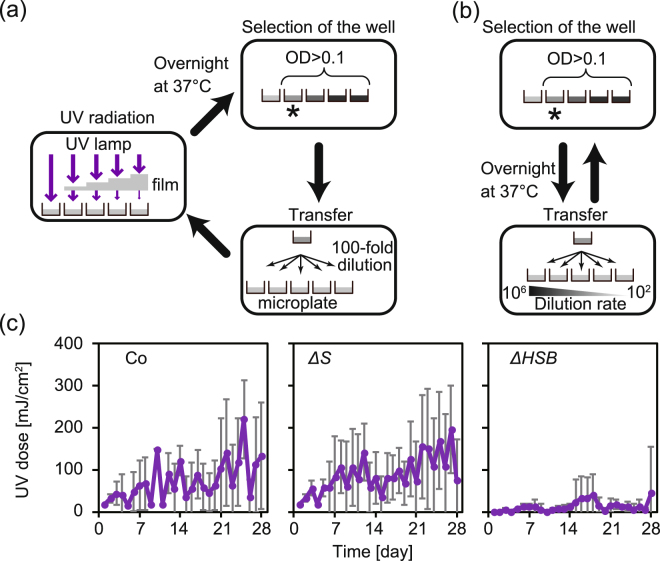
Evolution experiment with UV exposure. (**a**) Schematics of the evolution experiment with UV radiation. The overnight cultures were transferred to fresh media in multiple-well microplates after a 100-fold dilution (5 wells for each independent lineage). Subsequently, the cells were exposed to UV irradiation through UV cut films so that each well was exposed to different UV doses. The cell cultures were incubated overnight at 37 °C. The wells which were exposed to the largest UV dose (indicated by an asterisk) were selected among the well-growing cultures (OD_595_ > 0.1). This cycle was repeated for 28 days. We established six independent replications for each strain. (**b**) Schematic of the rounds of the evolution experiment without UV irradiation. The overnight cultures of the selected wells were transferred to fresh media with different dilution rates (10^2^-, 10^3^-, 10^4^-, 10^5^-, and 10^6^-fold). The wells which had the largest dilution rates (indicated by an asterisk) were selected among the well-growing cultures (OD_595_ > 0.1). This cycle was repeated for 28 days. Six independent replications were established for each strain. (**c**) The time series of the UV doses. The mean values among the six lineages were plotted for each strain (Co, *ΔS*, and *ΔHSB* from left to right). The error bars represent standard deviations.

To test whether the UV-induced mutation rate could be kept equivalent among the different strains by keeping the survival fractions equivalent, we performed an evolution experiment by exposing bacterial cultures UV radiation for 28 days with the maximum dosage of UV which did not annihilate the population. The daily procedure included performing growth assays on the cultures, serial transferring the selected culture, and exposing them to UV irradiation (Fig. 2a). First, the growth assay was performed by measuring the optical density at 595 nm, OD_595_, of the overnight cultures on micro-well plates (100 μl/well, 6 lineages/strain, and 5 wells/lineage) with a plate reader. We selected the well which was exposed to the largest UV dose among the sufficiently growing cultures (OD_595_ > 0.1) within each lineage. Next, the selected culture was transferred into fresh medium (5 wells/lineage) after a 100-fold dilution. Subsequently, the 5 wells were exposed to different doses of UV and incubated overnight for the next cycle. The growth selection (OD_595_ > 0.1) tended to keep the survival fraction in response to the UV exposure equal when the viability in response to UV exposure changed during the evolution experiment. We also conducted the evolutionary experiment without UV irradiation as a control (Fig. 2b). In this experiment, the overnight cultures of the selected wells were transferred to fresh medium with different dilution rates. We selected the wells which had the largest dilution rates among the sufficiently growing cultures (OD_595_ > 0.1) to maximise the number of generations. These cycles were repeated for 28 days for both evolution experiments. The exposed UV doses were monitored as shown in Fig. 2c. Overall, the UV doses increased at the end (day 27 and 28) compared to the beginning (day 1 and 2) (Wilcoxon’s signed rank test*, p* < 0.05 for all strains). The results revealed almost similar time series between Co and *ΔS*, while *ΔHSB* maintained a lower UV dose relative to the others. This fact was consistent with the differences in UV sensitivity and viability among the ancestral strains.

### Evolutionary changes in viability and mutability in the presence of UV exposure

**Figure 3:**
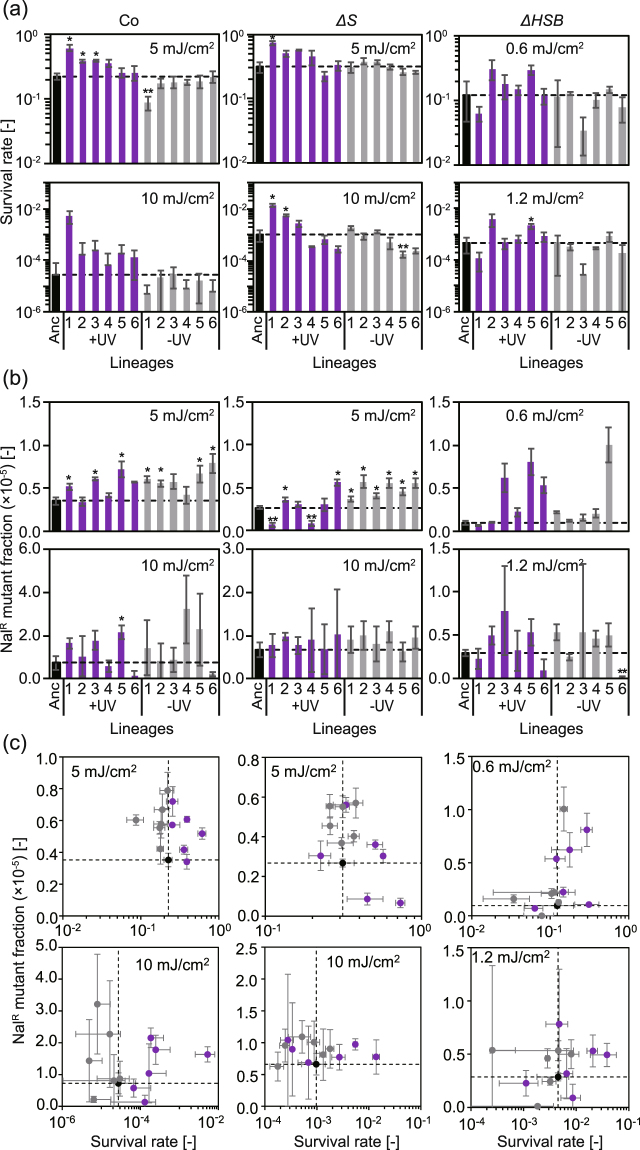
Viability and mutability of evolved strains in response to UV exposure. Survival rate (**a**) and Nal^R^ mutant fraction (**b**) in response to UV exposure are shown for the final populations in the evolution experiment and their ancestral strains. UV doses of 5 and 10 mJ/cm^2^ were used for Co and *ΔS*, while 0.6 and 1.2 mJ/cm^2^ were used for *ΔHSB*. The mean values were calculated for six lineages of each evolved strain. The error bars represent standard deviations. Asterisks indicate that the survival rates or mutation fractions significantly increased (single asterisks) or decreased (double asterisks) compared to the ancestors after evolution (*t*-test or Mann-Whitney U test, FDR < 0.05, see *Methods*). (**c**) Scatter plots of survival rates and Nal^R^ mutant fractions of the ancestral strains (black circles), the lineages with/without UV exposure during evolution experiment (purple/grey circles, respectively). The values were replotted from (**a**) and (**b**). The vertical/horizontal dashed lines indicate the values of the ancestral strains.

In agreement with the increase in UV doses during the evolution experiment, the survival rate in response to UV exposure (5 mJ/cm^2^ and 10 mJ/cm^2^) slightly but significantly (t-test, *p* < 0.05) increased in some lineages of all strains evolved in the presence of UV exposure (Fig. 3a, purple bars with asterisks). In contrast, this increase was rarely observed in the lineages evolved in the absence of UV exposure (Fig. 3a, grey bars). These results indicated that adaptive evolution toward UV tolerance occurred in some lineages in the presence of UV radiation. We also found that the survival rates varied between lineages in the presence of UV exposure, which was consistent with the large variance in UV dose identified during the evolution experiment. That is, many lineages did not increase their UV tolerance a significant amount in this short-term study, in particular *ΔS* and *ΔHSB*. Interestingly, mutability in response to UV exposure also tended to increase during the evolution experiment in the presence of UV exposure. In addition, this tendency was also found even in some lineages in the absence of UV exposure (Fig. 3b). That is, the mutability of the mutants selected in the populations did not decrease even though the viability increased (Fig. 3c), implying that possible error-correcting mechanisms didn’t improve during the evolution experiments. Therefore, the effect of UV tolerance on viability was not an evolutionary consequence of the improvement of possible error-correcting mechanisms to protect against UV-induced mutations.

**Figure 4:**
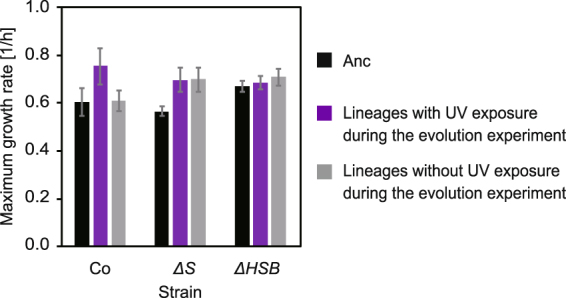
Growth characteristics of evolved strains. The final populations in the evolution experiment (the lineages with or without UV exposure on day 28) were compared with their ancestral strains. The bars for Anc were replotted from Fig. 1b for reference. The mean values were calculated for six lineages of each evolved strain. The error bars represent standard deviations. The maximum growth rates significantly increased compared to the ancestors after the evolution experiments (*t*-test, *p* < 0.05) except for the lineages of Co without UV exposure during the evolution experiments.

Growth rates can be a selective trait in evolution experiments regardless of UV exposure because there is growth selection after UV exposure even in evolution experiments in the presence of UV exposure (Fig. 1a). To examine this possibility, we compared the maximum growth rates of the evolved lineages in the absence of UV exposure with those of their ancestors (Fig. 4). As expected, the maximum growth rates of all strains evolved in the presence of UV exposure slightly increased after the evolution experiments (Mann-Whitney U test, *p* < 0.05). In addition, the lineages of two mutator strains also exhibited statistically significant increases in the maximal growth rates even in the absence of UV exposure (Mann-Whitney U test, *p* < 0.05). Only the lineages of Co without UV exposure during the evolution experiment exhibited no significant increase, indicating the accelerated growth adaptation caused by the higher mutation rate. The higher maximum growth rates of the lineages with UV exposure during the evolution experiments were almost similar to those of the mutator lineages without UV exposure, suggesting that no or only a small fraction of the UV-induced mutations contributed to the growth adaptation of the mutator lineages.

### Mutation accumulation during the evolution experiment with UV exposure

**Figure 5:**
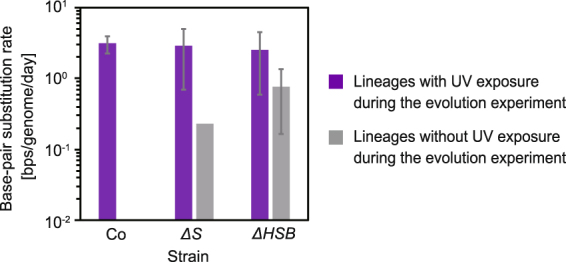
Base-pair substitution rate during the evolution experiments. The base-pair substitution rates were calculated using the number of accumulated mutations. The substitution rate for Co without UV exposure during the evolution experiment was below the detection limit. The error bars represent standard deviations (n = 6 for the lineages with UV, n = 2 for the lineages without UV).

Whole genome sequencing revealed the number of genomic mutations fixed within the populations (Tables 1 and S1). Using the number of synonymous substitutions, we calculated the accumulation rate of base-pair substitutions (BPSs) during the evolution experiments (Fig. 5). The accumulation rates in the absence of UV reflected the spontaneous mutation rate (Fig. 1a). The number of synonymous mutations in Co in the absence of UV exposure was too small to obtain a reliable mutation rate, which was consistent with the wild-type value in a previous study^3^. The accumulation rates of the mutators in the absence of UV were detectable and, as expected, were higher compared to that of Co^6^. We found that the accumulation rate of *ΔHSB* was slightly higher than that of *ΔS*, which differed from the values obtained from the fluctuation test (Fig. 1a). This difference may reflect the difference in the number and/or locus of marker genes between the two methods, as discussed previously^6^. That is, the fluctuation test monitored a few mutations on a few genomic loci, while genomic sequencing monitored many mutations over the genome. Another possible reason includes subtle differences in the mutational spectrum between the two mutators. The resistant mutants might have different mutations in these strains, which could result in the different mutation rates obtained from the two assessments^18^. Thus, the accumulation rates during the evolution experiment without UV exposure and the spontaneous mutation rates varied for the different genetic backgrounds as expected. Next, we estimated the accumulation rates in the presence of UV exposure. We found no significant correlation between the number of synonymous substitutions and advantageous traits during the evolution experiment such as survival rate in response to UV (ρ = 0.04 and 0.27 for 5 mJ/cm^2^ and 10 mJ/cm^2^, respectively, *p* > 0.05) or maximal growth rate (ρ = 0.04, *p* > 0.05), indicating that few of the accumulated mutations were advantageous. We noted that the UV-induced mutations might occur intermittingly in contrast to spontaneous mutations. We found almost the same accumulation rates in the presence of UV exposure for all strains (ANOVA, *F*(2,14) = 0.15, *p* = 0.86). These accumulation rates were higher than those in the absence of UV exposure (26-fold and 3.4-fold increase for *ΔS* and *ΔHSB*, respectively). These results indicated that the UV-induced mutagenesis was dominant relative to the spontaneous mutation rates and that the accumulation rates could be equivalent (Fig. 5) regardless of differences in the exposed UV dose (Fig. 2c), viability in response to UV (Fig. 1c–e), and/or spontaneous mutation rates (Fig. 1a).

**Table 1:** The number of BPSs in the coding regions.

Strain	UV treatment*	Lineage	*N*_*RNA*_	*N*_*syn*_	*N*_*nsyn*_	Total	dN/dS
Co	+	1	0	36	73	109	0.96
Co	+	2	1	24	41	66	0.93
Co	+	3	1	28	66	95	1.21
Co	+	4	NA	NA	NA	NA	NA
Co	+	5	1	26	32	59	0.69
Co	+	6	0	17	12	29	0.31
Co	−	1	0	0	1	1	NA
Co	−	2	0	0	1	1	NA
*ΔS*	+	1	0	42	78	120	0.94
*ΔS*	+	2	1	36	60	97	0.82
*ΔS*	+	3	0	2	5	7	1.48
*ΔS*	+	4	0	3	8	11	1.47
*ΔS*	+	5	1	36	74	111	0.94
*ΔS*	+	6	0	22	48	70	1.14
*ΔS*	−	1	0	0	3	3	NA
*ΔS*	−	2	0	2	2	4	0.53
*ΔHSB*	+	1	0	29	59	88	1.06
*ΔHSB*	+	2	1	25	61	87	1.10
*ΔHSB*	+	3	4	43	76	123	0.90
*ΔHSB*	+	4	0	3	4	7	0.79
*ΔHSB*	+	5	0	26	58	84	1.17
*ΔHSB*	+	6	0	1	10	11	5.93
*ΔHSB*	−	1	1	3	6	10	1.10
*ΔHSB*	−	2	0	10	8	18	0.41

^*^Plus and minus signs represent the lineages with or without UV, respectively.

*N*_*RNA*_ represents the number of base-pair substitutions in RNA genes.

*N*_*syn*_ represents the number of synonymous substitutions.

*N*_*nsyn*_ represents the number of nonsynonymous substitutions.

### Mutational spectrum and local sequence context of the UV-induced mutations

**Figure 6:**
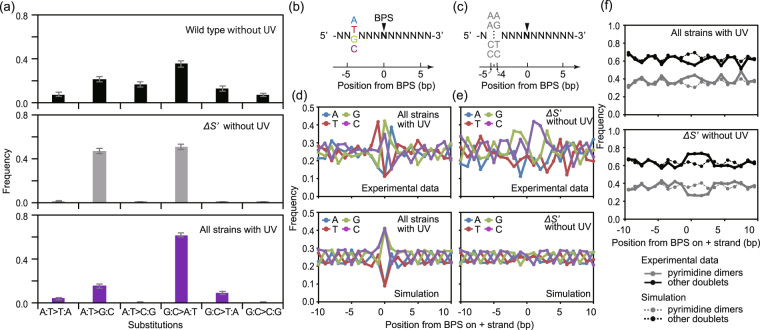
Spectra and local sequence context of BPSs. (**a**) The frequency of each type of synonymous substitution. The spectrum for the spontaneous mutations for the wild type (top) and *mutS*-defective strain (middle), denoted as *ΔS’*, were obtained from previous studies^3,6^. The spectrum for the UV-induced mutations was calculated by combining all strains (bottom). Error bars represent standard deviations from 1,000 Monte Carlo simulations for each dataset. These distributions significantly differed from each other (Kolmogorov–Smirnov test, *p* < 0.05). The frequency of BPS was measured for each singlet nucleotide (**b**,**d**,**e**) or doublet (**c**,**f**) which appeared around the BPS (−10 bp to 10 bp). (**b**) and (**c**) show the schematics of the sequences (N means any nucleotide) around a BPS (indicated by triangles), where the frequency of BPS was calculated for each nucleotide (A, T, G, and C) −4 bp from the BPS in (**b**) or in any doublet −4.5 bp from the BPS in (**c**). Simulations were based on the Monte Carlo method with the mutational spectra observed for each dataset.

The analysis of the synonymous substitutions that accumulated during the evolution experiments revealed a unique mutational spectrum (Fig. 6a and Table S2). The spectra of the spontaneous substitutions were previously explored for a wild type^3^ and *mutS*-defective mutator strain^6^ (Fig. 6a, top and middle). Contrary to the wide distributions for the wild type, the mutator strain exhibited two peaks at AT to GC and GC to AT and very small frequencies for the other substitutions (Fig. 6a, bottom). This transition-biased spectrum of the mutators was similar to that other mutator strains with deficient mismatch-repair and/or proofreading processes^3,6^. We also confirmed the similar spectra properties of the two mutators used in this study, *ΔS* and *ΔHSB*, even though there were fewer accumulated substitutions than the values reported in the previous studies (Table 1). Compared with the typical spectrum of mutators, the spectrum of the UV-induced synonymous substitutions for all strains, including Co, was broader. The fraction of GC to TA was still high, while the fractions of AT to TA, AT to GC, and GC to TA were at levels comparable to that of the wild type. That is, the UV-induced substitutions included not only transitions but also transversions similar to the spontaneous substitutions in the wild type.

We further explored the local sequence context of synonymous BPSs (Fig. 6b–e) to identify which single nucleotide within the neighbouring sequence (−10 bp to 10 bp) is likely to appear with each BPS. To detect some specific sequence contexts of BPSs, Monte Carlo simulations were performed as a null hypothesis based on no biased context, where BPSs were generated in the genomes at random with the corresponding mutational spectra. The frequent occurrence of UV-induced BPSs at a G or C reflects the corresponding mutational spectrum (Fig. 6a, and green and purple lines at 0 bp in Fig. 6d). In addition, these BPSs were likely to occur at the 5ʹ side of A or the 3ʹ side of T on a given DNA sequence (red line at −1 bp and blue line at +1 bp in Fig. 6d). Thus, the motif sequences “5ʹ-TC-3ʹ” or “5ʹ-TG-3ʹ” were prone to be mutated by UV exposure. The former motif is a dipyrimidine, which is consistent with the evidence that dipyrimidine sites can be a mutation hot spot since they frequently form DNA lesions (pyrimidine dimers) in response to UV exposure and are likely to introduce BPSs, in particular C to T, at the damaged sites, as reviewed previously^19^. In contrast, we did not detect these simple motifs for spontaneous synonymous substitutions in the *mutS*-defective strain (Fig. 6e), which was used in a previous study^6^. We examined the simulation note that +2 bp side of G, −2 bp side of C, 3ʹ side of G, or 5ʹ side of C might at least be an error-prone motif.

Next, we explored how dipyrimidine sites, two adjacent pyrimidines such as TT, relate to the BPSs within a short range of sequences (Fig. 6f). Sixteen possible DNA doublets were divided into two types: eight dipyrimidines (TT, TC, CT, CC, AA, AG, GA, and GG) and eight doublets. UV-induced mutations occurred at the dipyrimidine sites (solid grey line in Fig. 6f top) about 1.6-fold as often as expected based on a no local sequence context (broken grey line). In contrast, spontaneous mutations in the *mutS*-defective strain occurred at dipyrimidine sites less often than expected (Fig. 6f, bottom). More importantly, mutations also frequently occurred at the non-dipyrimidine sites in the presence or absence of UV exposure (solid black lines). These results indicated that UV-induced mutations occurred at various sites in the genome even though there was slight bias according to the local sequence context.

### Evolutionary consequence of spontaneous mutation rates

**Figure 7:**
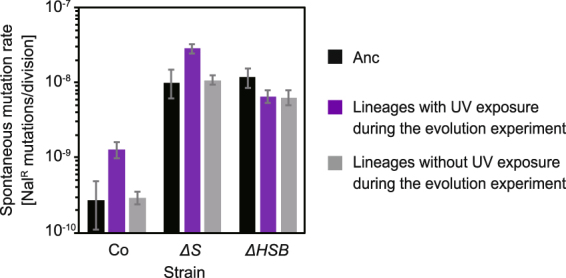
Spontaneous mutation rate of evolved strains. The spontaneous mutation rate was measured with a fluctuation test for the final populations in the evolution experiment and their ancestral strains. The bars for Anc were replotted from Fig. 1a for reference. The mean values were calculated for the six lineages of each evolved strain. The error bars represent 95% confidential intervals.

Interestingly, the spontaneous mutation rates increased for some lineages with UV exposure during the evolution experiment (Fig. 7). We measured the spontaneous mutation rates for the evolved strains using the fluctuation test in the absence of UV exposure. The spontaneous mutation rates of the lineages without UV exposure during the evolution experiment were almost steady. In contrast, the spontaneous mutation rates for the evolved strains of Co and *ΔS* which were transferred in the presence of UV exposure during the evolution experiment increased a few fold compared to their respective ancestral strains. Importantly, the increased spontaneous mutation rates were still far below the mutation rate during the evolution experiments with UV exposure (Figs 1a and 5). This implies that the advantage of low spontaneous mutation rate by the error correcting systems was nearly diminished under the higher endogenous mutation rate by the UV exposure. That is, the increase in the spontaneous mutability was considered to be derived from neutral evolution rather than adaptive evolution^20^. The spontaneous mutation rates for *ΔHSB* were almost the same among all conditions. This steadiness was rationale since the genetic mutation rate, monitored by BPSs, of this strain had already been higher than that of the other strains (Fig. 5, grey bars) and was only a fraction of the UV-induced mutation rate. Thus, these results possibly explain the increase in the spontaneous mutation rate and indicated that it can be prevented by negative selection against harmful mutations.

## Discussion

In this study, we explored both the frequency and spectrum of UV-induced mutations at the genomic level. We found that the mutation rate could be increased by UV exposure to a level hundreds times that of the typical spontaneous mutation rate with proficient error-correcting mechanisms. This frequency was comparable to or even greater than that of mismatch repair-deficient mutator strains, indicating that the upper limit of the mutability of UV radiation is very high even though UV exposure may introduce other toxic effects unrelated/related to mutations. The mutational spectrum of the UV-induced mutations was narrower than that of the spontaneous mutations in the wild type but broader than that of the mutators. Thus, UV exposure can compensate for some mutations which could not be increased in the mutators.

In conclusion, based on the genomic mutations accumulated in the presence of UV exposure, we demonstrated the broad spectrum and high upper limit of the frequency of occurrence in the UV-induced mutations. These results support the considerable contributions of UV as an extrinsic mutagen for generating diverse mutants rapidly both under natural and artificial conditions in the presence of massive UV exposure. This implies that evolution under research conditions without an extrinsic source of mutations might be different from that under natural or clinical conditions. For example, the probability of mutants gaining resistant to an antibiotic under these conditions might be higher or lower than expected based on the experiments under laboratory conditions.

The similar rates of mutation accumulation among the different genetic backgrounds implies there is a limit to mutation production per generation. The majority of the accumulated mutations in the lineages in the presence of UV exposure were caused by UV exposure. It is interesting that the rates of mutation accumulation in these lineages were quite similar even though the exposed doses varied among the lineages. This seeming contradiction can be explained by considering the survival rate in response to UV exposure. In our evolution experiments with UV exposure, the UV dosage in each round was determined for each lineage by considering the growth rate after each UV exposure and the survival rate in response to UV exposure. Here, the growth rates of the lineages were roughly similar. Therefore, the differences in UV dosage among the lineages were thought to be mainly caused by differences in the survivability to UV exposure. In the evolution round, a small UV dose was selected to maintain the daily propagation level in case the survivability to UV was low. Thus, the survival fraction after UV exposure, that is, the ratio of the final surviving cells after UV exposure in each round to the cells present before the UV exposure, was kept constant in order to maintain daily propagation by tuning the UV doses. This was consistent with the small UV doses used for the UV-sensitive strain, *ΔHSB*, relative to those used for the other strains. The reason why the number of accumulated mutations was the same when the survival fraction was the same among the lineages was that the *uvrB* gene is a part of a mutation repair system; the low survivability to UV exposure results from the high production rate of deleterious or lethal mutations caused by UV exposure. Accordingly, the number of these toxic mutations would be the same between the low survivability with a small UV dose and the high survivability with a large UV dose as long as the survival fraction was kept constant. Assuming that the fractions of the deleterious and lethal mutations in all mutations were the same for all strains, the number of accumulated mutations in the survivors would be the same for populations with the same survival fraction.

To determine how efficiently UV exposure can promote mutation accumulation, we assumed that UV irradiation causes at least two types of damage simultaneously in a cell population. One type includes both lethal and non-lethal mutations. Following conventional assumptions, we considered that the number of mutations per cell, *m*, follows a Poisson distribution with the rate parameter *λ*. The other type includes deleterious physiological side effects such as unrecoverable double-strand breaks in genomic DNA. For simplicity, we assume that the physiological damages are lethal and countable. In practice, the number of physiological damages per cell, *p*, also follows Poisson distribution with the rate parameter *γ*. For *m* mutations, their lethality follows a binomial distribution with the rate of lethal mutations (*l*). Then, the probability distribution at which a cell has *p* lethal physiological damages with *q* lethal mutations out of *m* mutations is given by

1 $$P(m,q,p)=\frac{{\lambda }^{m}{e}^{-\lambda }}{m!}(\begin{array}{c}m\\ q\end{array}){l}^{q}{(1-l)}^{m-q}\cdot \frac{{\gamma }^{p}{e}^{-\gamma }}{p!}.$$

We assume that the non-lethal mutations are neutral, i.e. they do not affect cell growth. Then, the survival rate (*S*) of the population after UV irradiation is written as

2 $$S=\sum _{m=0}^{\infty }P(m,q=0,p=0)={e}^{-(\lambda l+\gamma )}.$$

Note that the ratio of *λl* to the power exponent, i.e. *λl*/(*λl* + * γ*), indicates the mutation production efficiency of UV against cell death. We could not estimate *λ* directly because we could not measure the number of mutations for dead cells. Alternatively, we estimated *λ* by considering a relationship between *λ* and the average number of mutations per survival cell, *ρ*. Here, *ρ* is detectable by genomic sequencing for survival cells and is given by the average number of non-lethal mutations with non-lethal physiological damages per survival subpopulation divided by the number of survival cells as follows,

3 $$\rho =\frac{{N}_{tot}\sum _{m=0}^{\infty }m\cdot P(m,q=0,p=0)}{{N}_{tot}S}=\lambda (1-l).$$

*Ν*_*tot*_ is the total number of cells in a population. Thus, we obtained a relevant relationship, where *ρ* is equivalent to the nun-lethal fraction, 1−*l*, of *λ*. We estimated *S* = 10^−4^ in this experiment according to the growth rate (Fig. 4). *l* was set as ~0.1 because the dN/dS values were roughly 0.8 ~ 0.9 (Table 1), suggesting that about 10~20% of mutations were eliminated from the population by selection. We used *ρ* = 3.13 [bps/genome/day] (Fig. 5, Co). Introducing these rough estimates into equations (2) and (3) yields *λl* = 0.35 [lethal mutations/genome/day] and *γ* = 8.86 [lethal side effects/genome/day]. The efficiency of mutation production was given as *λl*/(*λl* + * γ*) = 0.038. This suggests that the dominant cause of cell death with UV exposure was not lethal genetic mutations but physiological side effects.

In order to discuss the general efficiency of UV radiation as a mutagen, we performed a similar estimate for spontaneous mutations as follows. Previously, we reported that the increase in the spontaneous mutation rate caused by deleting genes related to error correction also introduced a reduction in growth^6,8^. Therefore, we can also calculate the efficiency of mutation production due to the lack of error-correcting genes from the growth defects in the hyper-mutator strains. Let us consider that the lack of these genes also causes two types of lethal damage: lethal mutations and some lethal side effects. For simplicity, the mutations and the deleterious side effects occur every replication event in this case. That is, equation (1) is also applicable, and *S* in equation (2) indicates the rate of success for each self-replication event in this situation. The growth rate of the hyper-mutable strains (*μ*_*mut*_) is represented as follows,

4 $${\mu }_{mut}={\mu }_{WT}S={\mu }_{WT}{e}^{-(\lambda l+\gamma )},$$

where *μ*_*WT*_ represents the growth rate of the wild type strain with a sufficiently low mutation rate. Equation (4) was fitted to the corresponding data indicating that the growth rate decreased with the mutation rate (Fig. 2 in Ishizawa *et al*.^8^). Assuming that *γ* is proportional to *λ* and *l* is 0.1, we obtained *γ*/*λ* = 4.85 ± 2.72 (average ± standard error). Then, the efficiency of mutation production for the lack of error-correcting genes was calculated as *λl*/(*λl* + * γ*) = 0.020 (standard error interval was 0.013–0.045). Interestingly, this value is comparable to that of UV exposure, suggesting that the low efficiency of mutation production for UV exposure is not a specific property of UV light.

## Methods

### Bacterial strains

We used *E. coli* MDS42^21^ and two mutator strains, MDS42*ΔmutS::Cm*, and MDS42*ΔmutH*, *ΔmutS*,*ΔuvrB::Cm*. These stains were named Co, *ΔS*, and *ΔHSB*, respectively. *ΔS* and *ΔHSB* were constructed by combinatorial deletions of MMR genes (*mutS*, *mutH*) and a UV-resistant gene (*uvrB*). All deletions were examined by standardised λ-red homologous recombination using the pKD46 plasmid^8,22^. The chloramphenicol resistance gene, *Cm* ^*R*^, was employed repeatedly as a selection marker in each deletion. *Cm* ^*R*^ was amplified by PCR from the pKD32 plasmid^22^ with appropriate primers for each deletion (Table S3). To examine multiple deletions in *ΔHSB*, *Cm* ^*R*^ was removed by FLP-FRT recombination using the pCP20 plasmid prior to subsequent deletion experiments^22^.

### Culture conditions

We used a chemically defined medium, mM63, which contains 62 mM K_2_HPO_4_, 39 mM KH_2_PO_4_, 15 mM (NH_4_)_2_SO_4_, 2 μM FeSO_4_·7H_2_O, 15 μM thiamine hydrochloride, 203 μM MgSO_4_·7 H_2_O, and 22 mM glucose^23^. LB agar plates supplemented with 80 μg/ml nalidixic acid sodium salt (Sigma-Aldrich), denoted as LB + Nal plates, were used for fluctuation tests.

### Evolutionary experiment

Evolutionary experiments consisted of cycles of serial transfer and UV exposure. The bacterial cells were cultured in mM63 liquid medium in a 96-well microplate (100 μl/well). The cells were diluted 100 times with fresh mM63 and transferred to five wells (100 μl each) of a new microplate. The five wells were covered by a UV cut film with a different UV diming rate for each well and exposed to UV radiation under a germicidal lamp (GL-15, Panasonic). The UV cut film was made in-house by patching plastic sheets cut out from a standard clear file folder (40% UV cut per sheet). The UV diming rate was adjusted by overlaying the number of the plastic sheets (0, 2, 4, 6, and 8 for the five wells). Subsequently, the microplate was sealed and incubated with shaking at 37 °C for 1 day. After incubation, the optical density (OD) at 595 nm of the five wells was measured with a plate reader (Infinite F200 PRO, Tecan). The cells in the well whose UV dose was the highest among the wells with growing cells (OD_595_ > 0.1) was used for the next round. The UV exposure time was extended as the UV resistance of the cells increased. UV intensity was recorded using a UV dosimeter (UVA, UVC light meter, YK-37UVSD, Lutron Electronics Inc., USA) before exposure (typically 0.10–0.21 mW/cm^2^). Frozen stocks of the cells were prepared every seventh round. The UV doses of the start (averaege doses of day 1 and 2) and the end (averaege doses of day 27 and 28) of the experiments were statistical tested with Wilcoxon’s signed rank tests for each strain (n = 6) in order to check if the doses increased through the experiment.

### Measurement of maximal growth rate

Cells from frozen stocks were inoculated into 100 μl of mM63 broth and incubated at 37 °C for 12 hours. The growing cells were then diluted 100 times with fresh mM63 and transferred to multiple wells (10 wells for each evolved strain and 60 wells for each ancestral strain) in a 96-well microplate (100 μl/well). The microplate was shaken at 37 °C in the plate reader, and OD_595_ was measured every 15 minutes. The maximum growth rate [h^−1^] was obtained from the slopes of the growth curves during the exponential growth phase (OD_595_ = 0.01~0.06) according to the standard Malthusian growth model.

### Measurement of survival rate and mutant production rate in response to UV exposure

Glycerol-stock cells were inoculated into 5 ml of mM63 broth and incubated at 37 °C. The overnight cell culture was diluted with fresh mM63 broth (over 20 ml) to a concentration of 10^7^ cells/ml. Then, 5 ml of the culture was sampled, and the remaining 15 ml of the diluted culture was transferred to a petri dish and exposed to UV radiation under a germicidal lamp (5 mJ/cm^2^ for Co and *ΔS*, 0.6 mJ/cm^2^ for *ΔHSB*). Subsequently, 5 ml of the culture was sampled, and the remaining 10 ml of the culture was exposed to UV radiation again, after which 5 ml of the culture was sampled. These culture samples (three samples for each glycerol stock) were spread on mM63 agar plates and incubated at 37 °C for 2 days. The number of colonies was counted to determine the colony forming units (CFUs). Survival rate was calculated by dividing the CFUs of the UV-exposed cultures by the CFUs of the culture without UV treatment. We also prepared cell cultures with UV treatment in the same manner (three samples for each glycerol stock) to measure mutant production rate. The sampled cultures were transferred to test tubes (5 ml/tube) and shaken at 37 °C. The overnight cell cultures were analysed with a flow cytometer (FACS Canto™II, Becton, Dickson and Company) to measure the cell concentration. The cultures were spread on LB + Nal agar plates (80 μl/plate) and incubated at 37 °C for 2–3 days. Mutant production rate was calculated by dividing the number of colonies that appeared on the plate by the number of cells in 100 μl of inoculant. The ancestral and evolved values were compared after checking their normality and equality of variances for each data sets (Shapiro–Wilk test and F test at significant level *p* < 0.05). We used Student t test for the normal and homoscedastic data, Welch’s t test for the data which were normal but not homoscedastic and Mann-Whitney U test for the non-normal data. We adjusted the significance level so that FDR (False Discovery Rate) cutoff was 0.05 using “qvalue” R package^24^.

### Fluctuation test

The rates of mutation resulting in nalidixic acid resistance (Nal^R^) were measured with fluctuation tests^25^. Glycerol-stock cells were inoculated into 5 ml of mM63 broth and shaken at 37 °C. The overnight cell culture was analysed with a flow cytometer to obtain the cell concentration. The cells were transferred to fresh mM63 broth in 20 test tubes (5 ml each) with a cell concentration of 100 cells/ml. These tubes were shaken at 37 °C for 18 ~ 30 hours. The grown cultures were centrifuged at 5160 × *g* for 5 min at room temperature. The pellets were resuspended with the residual supernatant and spread on LB + Nal plates. These plates were incubated at 37 °C for 2 ~ 3 days, and the colonies that appeared were counted. The mutation rate was calculated using the MSS-maximum likelihood method^26^.

### Whole-genome sequencing

Genomic DNA was extracted using Wizard Genomic DNA Purification kits (Promega). DNA libraries were prepared as previously reported^6^. We performed multiplex analysis (typically 6 plex) with paired-end sequencing (251 bp) using a MiSeq Reagent Kit v2 and 500 cycles (Illumina). Raw sequences were processed by removing adaptor sequences, trimming bases with a quality below Q20 from the 3′ end of each read, and removing reads with lengths shorter than 40 bp, using cutadapt-1.4.1^27^ as previously described^6^. Using Burrows–Wheeler Aligner software (BWA), the reads were aligned onto the *E. coli* MDS42 reference chromosome (Accession: AP012306, the origin of NC_020518, GI: 471332236). Base pair substitutions (BPSs) were identified using SAMtools^28^ with default parameters, where the maximum read depth (-D option) was set to 500. For all samples, at least 99.8 percent of the genomic region was covered with read(s). The depth of coverage was (2.3 ± 0.5) × 100 (average ± sd). The called mutations with a value <100 for the Phred quality score^29,30^ were removed. Subsequently, BPSs with values <90% of the frequency of “mutant” reads were also removed. We regarded that the filtered mutations were dominant or fixed in the population.

### Monte Carlo simulations for the local sequence context of BPS

Synonymous BPSs were generated in the corresponding genome at random with the observed mutational spectra. We used the genome sequence of MG1655 for the *mutS*-defective strain, *ΔS’*, derived from MG1655^6^ and that of MDS42 for all strains evolved under UV. The simulation was examined for 10,000 trials for each dataset.

### Measurement of base-pair substitution rate during the evolution experiment

The rate of base pair substitution (genome^−1^day^−1^), *ρ*, was calculated using the following formula:

5 $$\rho =\frac{{N}_{syn}\cdot L}{F(syn)\cdot {L}_{CDS}\cdot D},$$

where *N*_*syn*_, *L*_*CDS*_, *L*, and *D* are the number of synonymous substitutions, the length of total coding DNA sequences per genome, genome size (3.98 Mbp for the MDS42 derivative strains), and the number of days of the evolution experiments, respectively. *F*_*(syn)*_ represents the probability that a substitution is synonymous when a substitution occurs and was calculated based on the codon usage and the probability of each substitution as described previously^6^.

### Calculating dN/dS values

The dN/dS value was estimated as the ratio of the number of nonsynonymous substitutions per nonsynonymous site, dN, and the number of synonymous substitutions per synonymous site, dS^6^. The values were calculated by the following equation:

6 $$dN/dS=\frac{{N}_{nsyn}}{{N}_{syn}}\cdot \frac{F(syn)}{1-F(syn)},$$

where, *N*_*syn*_ and *N*_*nsyn*_ represent the number of synonymous and nonsynonymoys substitutions shown in Table 1, respectively. *F*_*(syn)*_ is, as descrived above, a probability of what a occurred substitusion is synonymous.

## Electronic supplementary material

Supplementary Information 
